# Influenza and Up-to-Date COVID-19 Vaccination Coverage Among Health Care Personnel — National Healthcare Safety Network, United States, 2022–23 Influenza Season

**DOI:** 10.15585/mmwr.mm7245a5

**Published:** 2023-11-10

**Authors:** Jeneita Bell, Lu Meng, Kira Barbre, Emily Haanschoten, Hannah E. Reses, Minn Soe, Jonathan Edwards, Jason Massey, Gnanendra Reddy Tugu Yagama Reddy, Austin Woods, Matthew J. Stuckey, David T. Kuhar, Kayla Bolden, Heather Dubendris, Emily Wong, Theresa Rowe, Megan C. Lindley, Elizabeth J. Kalayil, Andrea Benin

**Affiliations:** ^1^Division of Healthcare Quality Promotion, National Center for Emerging and Zoonotic Infectious Diseases, CDC; ^2^Goldbelt C6, Chesapeake, Virginia; ^3^Lantana Consulting Group, East Thetford, Vermont; ^4^Chenega Enterprise Systems & Solutions, LLC, Chesapeake, Virginia; ^5^CACI International, Inc, Reston, Virginia; ^6^Immunization Services Division, National Center for Immunization and Respiratory Diseases, CDC.

**Summary**

**What is already known about this topic?**

CDC and the Advisory Committee on Immunization Practices recommend that health care personnel (HCP) receive an annual influenza vaccine and stay up to date with recommended COVID-19 vaccination.

**What is added by this report?**

During the 2022–23 influenza season, influenza vaccination coverage was 81% among HCP at acute care hospitals and 47% among those at nursing homes. Up-to-date COVID-19 vaccination coverage was 17% among HCP at acute care hospitals and 23% among those at nursing homes.

**What are the implications for public health practice?**

There is a need to promote evidence-based strategies to improve vaccination coverage among HCP. Tailored strategies might be useful to reach all HCP with recommended vaccines to protect them and their patients from vaccine-preventable respiratory diseases.

## Abstract

The Advisory Committee on Immunization Practices recommends that health care personnel (HCP) receive an annual influenza vaccine and that everyone aged ≥6 months stay up to date with recommended COVID-19 vaccination. Health care facilities report vaccination of HCP against influenza and COVID-19 to CDC’s National Healthcare Safety Network (NHSN). During January–June 2023, NHSN defined up-to-date COVID-19 vaccination as receipt of a bivalent COVID-19 mRNA vaccine dose or completion of a primary series within the preceding 2 months. This analysis describes influenza and up-to-date COVID-19 vaccination coverage among HCP working in acute care hospitals and nursing homes during the 2022–23 influenza season (October 1, 2022–March 31, 2023). Influenza vaccination coverage was 81.0% among HCP at acute care hospitals and 47.1% among those working at nursing homes. Up-to-date COVID-19 vaccination coverage was 17.2% among HCP working at acute care hospitals and 22.8% among those working at nursing homes. There is a need to promote evidence-based strategies to improve vaccination coverage among HCP. Tailored strategies might also be useful to reach all HCP with recommended vaccines and protect them and their patients from vaccine-preventable respiratory diseases.

## Introduction

Vaccination of health care personnel (HCP) is a critical strategy to minimize transmission of infection in health care settings (*1*,*2*). HCP are at high risk for work-related exposure to viruses such as influenza and SARS-CoV-2 but are less likely to transmit these infections when they are vaccinated (*3*). The Advisory Committee on Immunization Practices (ACIP) recommends that HCP receive an annual influenza vaccine (*4*). ACIP also recommends that persons aged ≥6 months stay up to date with recommended COVID-19 vaccination.^†^ The Centers for Medicare & Medicaid Services (CMS) monitors the implementation of these recommendations by requiring health care facilities such as nursing homes and acute care hospitals to report influenza^§^ and COVID-19^¶^ vaccination coverage among HCP** to CDC’s National Healthcare Safety Network (NHSN). This study examined influenza and up-to-date COVID-19 vaccination coverage among HCP working in acute care hospitals and nursing homes during the 2022–23 influenza season.

## Methods

### Data Collection

Acute care hospitals and nursing homes report data to NHSN according to surveillance protocols for influenza and COVID-19 vaccination. Acute care hospitals and nursing homes began reporting COVID-19 vaccination among HCP in 2021; nursing homes were required to report influenza vaccination among HCP for the first time during the 2022–23 influenza season.^††^ To assess influenza vaccination coverage, facilities are required to report an annual count of HCP working in the facility for ≥1 day during an influenza season (October 1–March 31)^§§^ and the number of HCP who 1) received influenza vaccination, 2) had a medical contraindication to influenza vaccination, 3) declined vaccination, and 4) had unknown vaccination status. The protocol for COVID-19 vaccination coverage includes parallel data fields for COVID-19; however, data collection occurs at a different cadence. Nursing homes and acute care facilities report on schedules mandated by their respective regulatory programs at CMS. Nursing homes submit COVID-19 vaccination coverage weekly^¶¶^; acute care facilities submit ≥1 week of data per month.*** Both types of facilities report COVID-19 vaccination coverage data among HCP who were eligible to work in the facility ≥1 day during the reporting week.

### Data Analysis

To assess HCP vaccination coverage during the 2022–23 influenza season, analyses were conducted using influenza and up-to-date COVID-19 coverage data (specifically, up-to-date COVID-19 coverage data from the week ending March 26, 2023, or the last submitted week of data) reported to NHSN. NHSN defined up-to-date COVID-19 vaccination as the receipt of a bivalent booster dose or completion of a primary series within the previous 2 months (i.e., not yet eligible to receive a bivalent vaccine).^†††^ Facilities reporting data for both vaccine types and employing at least five HCP were included in the analysis. Pooled mean influenza and up-to-date COVID-19 vaccination coverage rates were calculated as the number of HCP who had received each recommended vaccine or vaccination series divided by the number of HCP working in all facilities. HCP reported to have a medical contraindication to COVID-19 vaccination were subtracted from the denominator of the up-to-date COVID-19 vaccination coverage calculation, to align with the measure adopted by CMS’s quality reporting programs.^§§§^ Coverage with each vaccine was calculated for HCP working at each facility type (nursing home or acute care hospital). Results were further stratified by employment category (employee, licensed practitioner, and student or volunteer); rural-urban classification (rural or urban)^¶¶¶^; county-level social vulnerability index (SVI) tertile****; facility size tertile^††††^; state; and U.S. region.^§§§§^ Counties in a lower SVI tertile are less socially vulnerable than are those in an upper SVI tertile. All analyses were conducted using SAS (version 9.4; SAS Institute). This activity was reviewed by CDC, deemed not research, and was conducted consistent with applicable federal law and CDC policy.^¶¶¶¶^

## Results

### Influenza Vaccination Coverage

Among approximately 8.4 million HCP working in 4,057 acute care hospitals, influenza vaccination coverage was 81.0% overall (Table 1); coverage was lowest (67.2%) among nonemployee licensed practitioners and was substantially higher among employees (83.1%) and nonemployee students and volunteers (85.2%). Among HCP working in acute care hospitals, influenza vaccination coverage was highest in the Midwest (84.7%) and lowest in the Pacific region (74.4%).

**TABLE 1:** Pooled mean influenza vaccination coverage among health care personnel working at acute care hospitals and nursing homes, by facility type — National Healthcare Safety Network, United States, October 1, 2022–March 31, 2023*

Characteristic	Influenza vaccination coverage							
	Nursing homes				Acute care hospitals			
	No. of facilities	No. of vaccinated HCP	No. of HCP	Coverage, %	No. of facilities	No. of vaccinated HCP	No. of HCP	Coverage, %
**Total**	**13,794**	**956,149**	**2,030,770**	**47.1**	**4,057**	**6,854,771**	**8,465,804**	**81.0**
**Staff member type**								
Employee	13,794	844,380	1,832,394	46.1	4,054	5,245,329	6,315,763	83.1
Nonemployee licensed practitioner	11,365	61,060	110,432	55.3	3,695	828,669	1,234,011	67.2
Nonemployee student or volunteer	4,500	50,709	87,944	57.7	3,447	780,773	916,030	85.2
**Facility size^†^**								
Small	4,573	156,855	327,271	47.9	1,352	351,836	455,343	77.3
Medium	4,614	269,800	585,075	46.1	1,352	1,268,677	1,648,273	77.0
Large	4,607	529,494	1,118,424	47.3	1,353	5,234,258	6,362,188	82.3
**Urbanicity** ^§^								
Rural	3,817	191,508	426,368	44.9	1,173	659,881	824,714	80.0
Urban	9,977	764,641	1,604,402	47.7	2,884	6,194,890	7,641,090	81.1
**Social vulnerability index** ^¶^								
Low	4,724	333,282	660,593	50.5	1,228	2,030,870	2,437,031	83.3
Medium	4,605	332,622	725,843	45.8	1,341	2,463,062	3,086,676	79.8
High	4,463	290,129	644,194	45.0	1,487	2,360,245	2,941,443	80.2
Region**								
Midwest	4,476	247,750	584,925	42.4	1,034	1,747,029	2,061,455	84.7
Mountain	484	38,160	64,260	59.4	200	330,765	398,865	82.9
Northeast	2,291	250,904	436,621	57.5	573	1,333,833	1,620,573	82.3
Pacific	1,421	126,090	206,518	61.1	471	854,017	1,148,524	74.4
South	5,122	293,245	738,446	39.7	1,779	2,589,127	3,236,387	80.0

**Abbreviation**: HCP = health care personnel.

* Each facility reported summary influenza vaccination data among HCP working in the facility for ≥1 day during October 1, 2022–March 31, 2023. Up-to-date COVID-19 vaccination coverage was reported to National Healthcare Safety Network each week; data from the week ending March 26, 2023, or the last submitted week of data, were used for analysis.

^†^ Facility size was calculated separately for acute care hospitals and nursing homes and was based on the tertile distribution of the total number of staff members per facility.

^§^ https://www.cdc.gov/nchs/data_access/urban_rural.htm

^¶^ https://www.atsdr.cdc.gov/placeandhealth/svi/index.html

** *South*: Alabama, Arizona, Arkansas, Delaware, District of Columbia, Florida, Georgia, Kentucky, Louisiana, Maryland, Mississippi, New Mexico, North Carolina, Oklahoma, South Carolina, Tennessee, Texas, Virginia, and West Virginia; *Midwest*: Illinois, Indiana, Iowa, Kansas, Michigan, Minnesota, Missouri, Nebraska, North Dakota, Ohio, South Dakota, and Wisconsin; *Mountain*: Colorado, Idaho, Montana, Nevada, Utah, and Wyoming; *Pacific*: Alaska, California, Hawaii, Oregon, and Washington; *Northeast*: Connecticut, Maine, Massachusetts, New Hampshire, New Jersey, New York, Pennsylvania, Rhode Island, and Vermont.

Among approximately 2.0 million HCP working in 13,794 nursing homes, influenza vaccination coverage was 47.1% overall; coverage was lowest among employees (46.1%) and substantially higher among nonemployee licensed practitioners (55.3%) and nonemployee students and volunteers (57.7%). Among HCP working in nursing homes, influenza vaccination coverage was highest in the Pacific region (61.1%) and lowest in the South (39.7%). Influenza vaccination coverage among HCP was similar across facility size, urban-rural status, and SVI for both nursing homes and acute care hospitals. Nursing homes in six states reported influenza vaccination coverage of ≥75% among HCP, whereas this level of coverage was reported in acute care hospitals in 40 states (Figure) (Supplementary Table, https://stacks.cdc.gov/view/cdc/134928).

**FIGURE:**
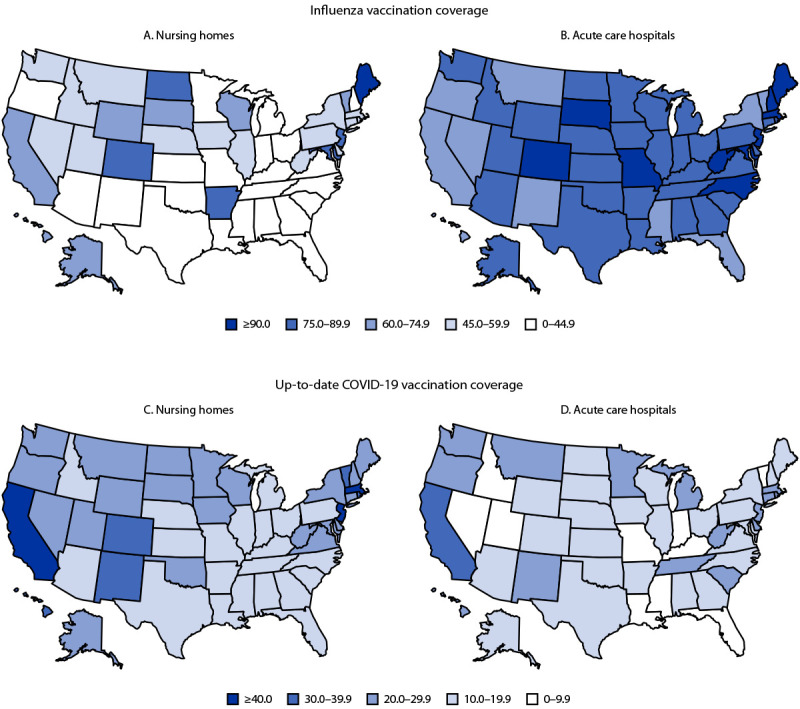
Percentage of pooled mean influenza vaccination coverage (A and B) and up-to-date COVID-19 vaccination*^,^^†^ coverage (C and D) among health care personnel working at nursing homes (A and C) and acute care hospitals (B and D), by facility type and U.S. state — National Healthcare Safety Network, United States, October 1, 2022–March 31, 2023 * Up-to-date COVID-19 vaccination coverage was defined by the National Healthcare Safety Network during the study period as the receipt of a bivalent booster dose, completion of a primary series, or receipt of a monovalent booster dose within the previous 2 months. ^†^ Each facility reported summary influenza vaccination data among health care personnel working in the facility for ≥1 day during October 1, 2022–March 31, 2023. Up-to-date COVID-19 vaccination coverage was reported to the National Healthcare Safety Network each week; data from the week ending March 26, 2023, or the last week of submitted data, were used for analysis.

### Up-to-Date COVID-19 Vaccination Coverage

Among approximately 7.7 million HCP working in 4,057 acute care hospitals, up-to-date COVID-19 vaccination coverage was 17.2% overall (Table 2) and was highest in the Pacific region (28.9%) and lowest in the Mountain region (9.1%). No substantial differences by staff member type or urbanicity were observed.

**TABLE 2:** Pooled mean up-to-date COVID-19 vaccination coverage* among health care personnel working at nursing homes and acute care hospitals, by facility type — National Healthcare Safety Network, United States, October 1, 2022–March 31, 2023^†^

Characteristic	Up-to-date COVID-19 vaccination coverage							
	Nursing homes				Acute care hospitals			
	No. of facilities	No. of vaccinated HCP	Total no. of HCP	Coverage, %	No. of facilities	No. of vaccinated HCP	Total no. of HCP	Coverage, %
**Total**	**13,794**	**376,837**	**1,652,744**	**22.8**	**4,057**	**1,328,820**	**7,725,167**	**17.2**
**Staff member type**								
Employee	13,794	341,672	1,523,365	22.4	4,051	1,029,896	5,879,220	17.5
Nonemployee licensed practitioner	10,006	25,372	89,975	28.2	3,496	199,869	1,203,932	16.6
Nonemployee student or volunteer	3,522	9,793	39,404	24.9	3,148	99,055	642,015	15.4
**Facility size** ^§^								
Small	4,573	76,018	309,005	24.6	1,352	70,547	448,596	15.7
Medium	4,614	116,077	494,368	23.5	1,352	228,176	1,540,690	14.8
Large	4,607	184,742	849,371	21.8	1,353	1,030,097	5,735,881	18.0
**Urbanicity** ^¶^								
Rural	3,817	60,121	343,954	17.5	1,173	109,089	745,548	14.6
Urban	9,977	316,716	1,308,790	24.2	2,884	1,219,731	6,979,619	17.5
Social vulnerability index**								
Low	4,724	125,753	544,753	23.1	1,228	415,883	2,247,508	18.5
Medium	4,605	130,598	591,079	22.1	1,341	472,906	2,816,438	16.8
High	4,463	120,463	516,798	23.3	1,487	439,848	2,660,648	16.5
**Region** ^††^								
Midwest	4,476	91,258	475,948	19.2	1,034	291,758	1,811,254	16.1
Mountain	484	13,227	49,615	26.7	200	33,396	365,336	9.1
Northeast	2,291	101,633	381,642	26.6	573	289,194	1,515,844	19.1
Pacific	1,421	70,245	172,738	40.7	471	308,765	1,066,996	28.9
South	5,122	100,474	572,801	17.5	1,779	405,707	2,965,737	13.7

**Abbreviations**: HCP = health care personnel.

* COVID-19 up-to-date coverage was defined by National Healthcare Safety Network during the study period as the receipt of a bivalent booster dose or completion of a primary series or receipt of a monovalent booster dose within the previous 2 months.

^†^ Each facility reported summary influenza vaccination data among HCP working in the facility for ≥1 day during October 1, 2022–March 31, 2023. Up-to-date COVID-19 vaccination coverage was reported to National Healthcare Safety Network each week; data from the week ending March 26, 2023, or the last submitted week of data, were used for analysis.

^§^ Facility size was calculated separately for acute care hospitals and nursing homes and was based on the tertile distribution of the total number of staff members per facility.

^¶^ https://www.cdc.gov/nchs/data_access/urban_rural.htm

** https://www.atsdr.cdc.gov/placeandhealth/svi/index.html

^††^ *South*: Alabama, Arizona, Arkansas, Delaware, District of Columbia, Florida, Georgia, Kentucky, Louisiana, Maryland, Mississippi, New Mexico, North Carolina, Oklahoma, South Carolina, Tennessee, Texas, Virginia, and West Virginia; *Midwest*: Illinois, Indiana, Iowa, Kansas, Michigan, Minnesota, Missouri, Nebraska, North Dakota, Ohio, South Dakota, and Wisconsin; *Mountain*: Colorado, Idaho, Montana, Nevada, Utah, and Wyoming; *Pacific*: Alaska, California, Hawaii, Oregon, and Washington; *Northeast*: Connecticut, Maine, Massachusetts, New Hampshire, New Jersey, New York, Pennsylvania, Rhode Island, and Vermont.

Among approximately 1.6 million HCP working at 13,794 nursing homes, up-to-date COVID-19 vaccination coverage was 22.8% overall; coverage was highest among nonemployee licensed practitioners (28.2%) and lowest among employees (22.4%). Among HCP working in nursing homes, up-to-date COVID-19 vaccination coverage was highest among those working in the Pacific region (40.7%) and lowest among those working in the South (17.5%). Up-to-date COVID-19 vaccination was also substantially higher among HCP working at nursing homes in urban (24.2%) than in rural (17.5%) areas. No substantial differences in COVID-19 vaccination coverage among HCP by facility staff size or SVI were observed at either facility type. Up-to-date COVID-19 vaccination coverage was ≥20% among HCP working in nursing homes in 30 states but among HCP in acute care hospitals, approximately one half as many states (16) achieved this level of coverage (Supplementary Table, https://stacks.cdc.gov/view/cdc/134928).

## Discussion

During the 2022–23 influenza season, fewer than one quarter of HCP working in acute care hospitals and nursing homes were up to date with recommended COVID-19 vaccination, and fewer than one half of HCP working in nursing homes had received influenza vaccine. Coverage varied by geographic region, health care facility type, employment category, and urbanicity. Recent reports indicate that influenza and COVID-19 vaccination coverage among HCP has declined during the COVID-19 pandemic (*5*). During the 2017–18 and 2018–19 influenza seasons, influenza vaccination coverage among HCP in acute care hospitals was 88.6% and 90.0%, respectively (*6*). From November 2021 to June 2023, CMS required all HCP at CMS-certified facilities to be vaccinated for COVID-19*****; this requirement likely contributed to COVID-19 primary series vaccination coverage reaching 94.3% among HCP in nursing homes (*7*) and 91.2% among those at acute care hospitals (*5*). The current findings suggest that factors associated with low vaccination coverage might have been exacerbated by the COVID-19 pandemic and compounded by emerging concerns such as vaccine fatigue (*8*) and other as yet unidentified factors.

In this analysis, up-to-date COVID-19 vaccination coverage was higher among HCP working in nursing homes than among those working in acute care hospitals. CMS requires nursing homes to report weekly up-to-date COVID-19 vaccination status among HCP and publishes weekly results on a public-facing website^†††††^; this might have resulted in higher coverage among HCP in nursing homes. CDC also worked with nursing homes to facilitate access to vaccination for both patients and staff members,^§§§§§^ which might have also improved coverage.

This report identified low up-to-date COVID-19 vaccination coverage among HCP in both acute care hospitals and nursing homes and low influenza vaccination coverage among HCP in nursing homes, both important threats to patient health and safety that need to be addressed. Implementation of vaccination recommendations for HCP has been a long-standing challenge for the public health and health care sectors. In an effort to improve vaccination coverage among HCP, health care facilities and federal and state governments have implemented interventions including jurisdiction-wide and facility-wide vaccination mandates (*7**,**9*). Mandates for HCP to receive influenza vaccination have been in place since before the COVID-19 pandemic and might contribute to the high vaccination rates reported to NHSN. However, such mandates might not be easily enforceable among nonemployee HCP in acute care hospitals, among whom coverage with both vaccines was lower than that among employees. Compared to influenza vaccines, COVID-19 vaccines are newer, and availability can be more sporadic; therefore, facilities do not have as much experience promoting vaccination and might not have the ability to conduct mass vaccination events. This might have contributed to lower COVID-19 vaccination coverage. Further, given the variations in vaccination coverage by region and urbanicity, campaign strategies tailored by region and focusing on rural areas might have the potential to increase vaccination coverage.

### Limitations

The findings in this report are subject to at least four limitations. First, influenza vaccination and up-to-date COVID-19 vaccination coverage rates were reported separately using different definitions of total HCP working within the facility. Whether the same personnel are represented in seasonal influenza vaccination coverage counts and weekly COVID-19 vaccination counts is unknown. This nuance limits the direct comparability of coverage with the two vaccines; therefore, statistical comparisons of vaccination coverage were not conducted. Second, this report includes data reported by facilities on behalf of HCP, which could have resulted in underestimates of vaccination acquired outside the health care facility, particularly by HCP not employed directly by the reporting facility. Third, vaccination coverage could not be stratified by recent history of SARS-CoV-2 infection. CDC recommendations state that persons may consider delaying an updated vaccine by 3 months after infection. Therefore, some persons might not have considered themselves eligible for vaccination, leading to an underestimate of COVID-19 vaccination coverage. Finally, this analysis was conducted using aggregate data reported to NHSN at the facility level. Therefore, vaccination coverage could not be stratified by person-level covariates that might have enabled an assessment of potential differences, such as age, race, and ethnicity. 

### Implications for Public Health Practice

Closely monitoring influenza and up-to-date COVID-19 vaccination coverage among HCP might help facilitate evaluation of effective implementation of vaccination promotion strategies.^¶¶¶¶¶^ Studies are needed to identify additional factors associated with low vaccination coverage and approaches to improve coverage among HCP, with particular attention to geographic region, health care facility type, and employment category. Understanding these factors and promoting evidence-based strategies to increase vaccination coverage among HCP, such as making vaccines free and accessible at work (*10*), might allow for targeted interventions to improve coverage during future respiratory virus seasons. HCP should receive annual influenza vaccines and remain up to date with recommended COVID-19 vaccination to protect themselves and their patients from vaccine-preventable diseases.

## Footnotes

* These authors contributed equally to this report.

†
https://www.cdc.gov/coronavirus/2019-ncov/vaccines/stay-up-to-date.html

§
https://www.cdc.gov/nhsn/faqs/vaccination/faq-influenza-vaccination-summary-reporting.html

¶
https://www.cdc.gov/nhsn/pdfs/hps/covidvax/protocol-hcp-508.pdf

**
https://www.cdc.gov/nhsn/forms/instr/COVIDVax.Staff_.Revised.TOI_.MAY2022-508.pdf

††
https://www.federalregister.gov/documents/2022/08/03/2022-16457/medicare-program-prospective-payment-system-and-consolidated-billing-for-skilled-nursing-facilities

§§
https://www.govinfo.gov/content/pkg/FR-2011-08-18/pdf/2011-19719.pdf

¶¶
https://www.federalregister.gov/documents/2021/05/13/2021-10122/medicare-and-medicaid-programs-covid-19-vaccine-requirements-for-long-term-care-ltc-facilities-and

***
https://www.federalregister.gov/documents/2021/08/13/2021-16519/medicare-program-hospital-inpatient-prospective-payment-systems-for-acute-care-hospitals-and-the?form

††† NHSN defines up-to-date vaccination for surveillance purposes at the start of each quarter; the definition has been updated since the study was conducted. Bivalent COVID-19 vaccines are no longer recommended or available. https://www.cdc.gov/nhsn/pdfs/hps/covidvax/UpToDateGuidance-508.pdf

§§§
https://www.cdc.gov/nhsn/pdfs/nqf/covid-vax-hcpcoverage-rev-2023-508.pdf

¶¶¶
https://www.cdc.gov/nchs/data_access/urban_rural.htm

****
https://www.atsdr.cdc.gov/placeandhealth/svi/index.html

†††† Facility size was calculated separately for acute care hospitals and nursing homes and was based on the tertile distribution of the total number of staff members per facility.

§§§§ *South*: Alabama, Arizona, Arkansas, Delaware, District of Columbia, Florida, Georgia, Kentucky, Louisiana, Maryland, Mississippi, New Mexico, North Carolina, Oklahoma, South Carolina, Tennessee, Texas, Virginia, and West Virginia; *Midwest*: Illinois, Indiana, Iowa, Kansas, Michigan, Minnesota, Missouri, Nebraska, North Dakota, Ohio, South Dakota, and Wisconsin; *Mountain*: Colorado, Idaho, Montana, Nevada, Utah, and Wyoming; *Pacific*: Alaska, California, Hawaii, Oregon, and Washington; *Northeast*: Connecticut, Maine, Massachusetts, New Hampshire, New Jersey, New York, Pennsylvania, Rhode Island, and Vermont.

¶¶¶¶ 45 C.F.R. part 46, 21 C.F.R. part 56; 42 U.S.C. Sect. 241(d); 5 U.S.C. Sect. 552a; 44 U.S.C. Sect. 3501 et seq.

*****
https://www.cms.gov/files/document/qso-23-02-all-expired.pdf

†††††
https://www.cms.gov/newsroom/news-alert/cms-makes-nursing-home-covid-19-booster-vaccination-data-available-online-increasing-transparency

§§§§§
https://archive.cdc.gov/www_cdc_gov/vaccines/covid-19/long-term-care/pharmacy-partnerships/jurisdictions.html#on-site

¶¶¶¶¶
https://www.cdc.gov/infectioncontrol/guidelines/healthcare-personnel/immunization.html
